# Supplementary material for: Cloud BioLinux: pre-configured and on-demand bioinformatics computing for the genomics community
Source: BMC Bioinformatics. 2012 Mar 19;13:42. doi: 10.1186/1471-2105-13-42 (PMC3372431; doi:10.1186/1471-2105-13-42)
Supplement: Additional file 1 — Supplementary 1 Cloud BioLinux software documentation in the form of a mini, self-contained website. Users need to download and uncompress the .zip file, and open through a web browser the "index.html" file available on the main directory. (ZIP 1823 kb). [file 1471-2105-13-42-S1.ZIP › Cloud-BioLinux-Package-Documentation/index.html]

Bio-Linux Software Documentation Pages

# Search Form

  

### Browse by Application/ Package Name

Please select...
2ft
6ft
aaindexextract
abiview
acdc
acdlog
acdpretty
acdtable
acdtrace
acdvalid
act
afetch
ajbad
ajfeatest
ajtest
aligncopy
aligncopypair
alistat
annotate
anomaly
antigenic
ape
arb
artemis
assembly-conversion-tools
atv
backtranambig
backtranseq
banana
baseml
basemlg
big-blast
bioconductor
BiodiversityR
biojava
bioperl
bioruby
biosed
bl2seq
blast+
blast2
blastall
blastcl3
blastclust
blastdbcmd
blastn
blastp
blastpgp
blastx
blixem
boxshade
BTmapqtl
btwisted
build-icm
bwa
caf2gap
caftools
cai
cap3
cd-hit
cd-hit-est
cdbfasta
cdbyank
chaos
charge
check
checktrans
chi2
chimeraslayer
chips
cirdna
clcsequenceviewer
clique
clmclose
clmconf
clmdist
clmformat
clmimac
clminfo
clmmate
clmmeet
clmresidue
clobb
clustalw
clustalx
cn3d
codcmp
codcopy
codeml
coderet
codon-usage
combineMUMs
compare-lists
complex
compseq
cons
consambig
consed
consense
contml
contrast
copymat
corbatest
cpgplot
cpgreport
cross\_match
cusp
cutgextract
cutseq
cytoscape
dan
dba
dbiblast
dbifasta
dbiflat
dbigcg
dbxfasta
dbxflat
dbxgcg
dbxstat
degapseq
demoalign
demofeatures
demolist
demostring
demostringnew
demotable
dendroscope
denoiser
density
descseq
dialign
diffseq
digest
distmat
dnacomp
dnadist
dnainvar
dnaml
dnamlk
dnamove
dnapars
dnapenny
dollop
dolmove
dolpenny
dotmatcher
dotpath
dotter
dottup
dotur
drawgram
drawtree
dreg
dust
dustmasker
edialign
einverted
Emap
embossdata
embossversion
emma
emowse
entrails
entret
Entrez
epestfind
eprimer3
Eqtl
equicktandem
est2genome
estscan
estwise
estwisedb
etandem
evolver
exact-tandems
extract
extractalign
extractfeat
extractseq
extract\_from\_pdb
factor
fasta
fastacmd
fastdnaml
fastf
fastm
FastQC
fasts
FastTree
fastx
fasty
featcopy
featreport
findkm
fitch
flalign
formatdb
formatrpsdb
freak
fuzznuc
fuzzpro
fuzztran
gap2caf
gap4
gaps
garlic
garnier
gcphrap
geecee
gendist
generate
genespring
genewise
genewisedb
get-len
get-putative
getorf
gff2ps
ggsearch
giep
glam2
glimmer2
glsearch
gromacs
gtop
HAPPY
helixturnhelix
histogramtest
hmmalign
hmmbuild
hmmcalibrate
hmmcalibrate-pvm
hmmconvert
hmmemit
Hmmer
hmmfetch
hmmindex
hmmpfam
hmmpfam-pvm
hmmsearch
hmmsearch-pvm
hmoment
htop
iep
impala
infoalign
infobase
inforesidue
infoseq
intconv
isochore
jalview
jaspextract
jaspscan
jMOTU
jprofilegrid
JZmapqtl
kitsch
lalign
lalign2list
Lamarc
lav2ps
lav2svg
lfasta
lindna
listor
long-orfs
LRmapqtl
lucy
makeblastdb
makemat
makembindex
makenucseq
makeprotseq
mapview
maq
marscan
maskambignuc
maskambigprot
maskfeat
maskseq
matcher
maxdLoad2
maxdview
mcl
mclfamily
mclfaq
mclpipeline
mcmctree
mcx
mcxarray
mcxassemble
mcxconvert
mcxdump
mcxmap
mcxsubs
mdust
megablast
megamerger
merger
mesquite
mgaps
migrate
MImapqtl
mira
mira-3rd-party
mix
mocca
mothur
move
mrbayes
mrbayes-multi
msbar
mspcrunch
MultiRegress
mummer
mummerplot
muscle
mview
mwcontam
mwfilter
needle
needleall
neighbor
netblast
newcoils
newcpgreport
newcpgseek
newseq
njplot
nohtml
noreturn
nospace
notab
notseq
nrdb
nthseq
nthseqset
nucmer
nucmer2xfig
ocount
octanol
oddcomp
OligoArray
omssa
origsplitter
origunion
palindrome
pamp
pars
partigene
pasteseq
patmatdb
patmatmotifs
patmattest
pedro
penny
pepcoil
pepinfo
pepnet
pepstats
peptidemapper
pepwheel
pepwindow
pepwindowall
pfmake
pfscale
pfscan
pfsearch
pftools
pfw
phrap
phred
phylip
Phyml
plalign
plfasta
plotcon
plotorf
polydot
postnuc
postpro
prank
preg
pregap4
prenuc
Preplot
prepro
prettyplot
prettyseq
prfx
priam
prima
primer3
primers
primersearch
printsextract
profit
PROmer
proml
PROmlk
prophecy
prophet
prosextract
prot4est
protdist
protpars
prss
Prune
psa2msa
pscan
psiblast
psiphi
psw
pswdb
ptof
ptoh
qiime
Qstats
QTLcart
QTL\_Cartographer
R
rasmol
Rcmdr
Rcross
rdp\_classifier
readseq
rebaseextract
recoder
redata
remap
repeat-match
restdist
restml
restover
restrict
retree
revseq
Rmap
roche2gap
rpsblast
rpsblast+
rpstblastn
Rqtl
run-glimmer2
run-mummer1
run-mummer3
samtools
seaview
seealso
seedtop
segmasker
seqboot
seqinfo
seqmatchall
seqret
seqretall
seqretallfeat
seqretset
seqretsetall
seqretsingle
seqretsplit
seqrettype
seqsplit
seqstat
sequin
sfetch
show-aligns
show-coords
show-tiling
showalign
showdb
showfeat
showorf
showpep
showseq
shuffle
shuffleseq
sibsim4
sigcleave
silent
sim4
sindex
sirna
sixpack
sizeseq
skipredundant
skipseq
spin
splitsource
splitstree
splitter
squint
sreformat
SRmapqtl
ssake
ssearch
stars
stretcher
stssearch
supermatcher
swat
syco
t-coffee
Taverna
taxblast
tblastn
tblastx
tcode
testplot
textsearch
tfastf
tfastm
tfasts
tfastx
tfasty
tfextract
tfm
tfscan
tmap
tqs
trace2dbest
tranalign
transeq
tree-puzzle
treedist
treeview
trev
trimest
trimseq
trimspace
twofeat
uclust
union
vectorstrip
velvet
water
weight
whichdb
windowmasker
wobble
wordcount
wordfinder
wordmatch
wossname
xsplits
yamap
yank
yn00
Zmapqtl

  

List all applications/packages

  
  
  

### Browse by Category

Please select...
Acd
Alignment
Clustering
Databases
Display
Edit
Enzyme\_kinetics
Feature\_tables
Hmm
Information
Menus
Microarray
Nucleic
Phylogeny
Pipelines
Protein
Statistics
Test
Utils

  

List all categories
